# Pathophysiological Mechanisms Linking COVID-19 and Acute Surgical Abdomen: A Literature Review

**DOI:** 10.3390/life15050707

**Published:** 2025-04-27

**Authors:** Andrei Modiga, Vlad-Olimpiu Butiurca, Cristian Marius Boeriu, Teodora Sorana Truta, Emilia Turucz, Vasile-Bogdan Halațiu, Ioana-Patricia Rodean, Paul Cristian Russu, Mircea Constantin Gherghinescu, Călin Molnar

**Affiliations:** 1 Doctoral School of Medicine and Pharmacy, George Emil Palade University of Medicine, Pharmacy, Sciences and Technology of Targu Mures, 540139 Targu Mures, Romania; andrei.modiga@umfst.ro; 2 Faculty of Medicine, George Emil Palade University of Medicine, Pharmacy, Sciences and Technology of Targu Mures, 540139 Targu Mures, Romania; cristian.boeriu@umfst.ro (C.M.B.); sorana.truta@umfst.ro (T.S.T.); emilia.turucz@umfst.ro (E.T.); bogdan.halatiu@umfst.ro (V.-B.H.); ioana.rodean@umfst.ro (I.-P.R.); paul.russu@umfst.ro (P.C.R.); mircea.gherghinescu@umfst.ro (M.C.G.); molnar.calin@yahoo.com (C.M.); 3 Clinical Emergency Department (UCPU-SMURD), County Emergency Clinical Hospital of Targu-Mures, 540136 Targu Mures, Romania; 4 General Surgery Clinic No. 1, County Emergency Clinical Hospital of Targu-Mures, 540136 Targu Mures, Romania

**Keywords:** acute surgical abdomen, COVID-19, inflammation, thrombosis

## Abstract

Acute surgical abdomen is characterized by intense, sudden abdominal pain due to intra-abdominal conditions requiring prompt surgical intervention. The coronavirus disease 2019 (COVID-19) pandemic has led to various complications related to the disease’s complex pathophysiological mechanisms, hence the hypothesis of COVID-19-induced acute abdominal surgical pathologies. The connection between acute surgical abdomen and COVID-19 involves two primary mechanisms. First, there is the presence of angiotensin-converting enzyme 2 (ACE2) receptors in multiple abdominal organs. This facilitates the cytokine storm through direct viral injury and inflammation. Second, the hypercoagulable state induced by severe acute respiratory syndrome coronavirus 2 (SARS-CoV-2) increases the thrombotic risk within abdominal vessels, which can subsequently lead to ischemia. ACE2 receptors are notably expressed in the gastric, duodenal, and rectal epithelium, with SARS-CoV-2 viral RNA and nucleocapsid proteins detected in these tissues. The inflammatory response results in significant endothelial damage, activating coagulation pathways that cause monocellular infiltration, lymphocytic inflammation, and uncontrolled coagulation. These findings highlight the need for further research to clarify how COVID-19 leads to acute abdominal pathologies. Understanding these mechanisms is vital for improving clinical management and patient outcomes during future health crises and in the aftermath of the pandemic.

## 1. Introduction

In the late months of the year 2019, an atypical pneumonia outbreak arose in the People’s Republic of China’s town of Wuhan. Later on, the causative agent was identified as being the novel coronavirus, part of the severe acute respiratory syndrome coronavirus family; hence, the name of the disease, coronavirus disease 2019 (COVID-19).

The great number of severe acute respiratory syndrome cases, the rapid global expansion, and the rapidly increasing numbers of deaths due to COVID-19 led the World Health Organization (WHO) to declare a global pandemic, starting in March 2020 [1].

According to data provided by Johns Hopkins University in the United States, as of April 2020, almost 2.5 million total confirmed cases of COVID-19 had been reported in over 205 nations and territories, resulting in about 170,400 fatalities [2].

Early investigations indicated that severe acute respiratory syndrome coronavirus 2 (SARS-CoV-2) likely originated in bats, while the intermediary host between the bat reservoir and the human remains unknown. COVID-19 is transmitted from person to person mostly through droplets and/or close contact between an infected and a healthy person. Although the virus has been found in affected people’s tears and stool, it is unknown if the disease spreads through the oral, fecal, or conjunctival pathways [3]. Patients with COVID-19 may be asymptomatic carriers or may experience symptoms ranging from mild to severe, including respiratory or multiorgan failure. Patients commonly report fever, dry cough, myalgia, tiredness, and dyspnea. Other symptoms include diarrhea, abdominal pain, altered mental status, active cough, pleuritic chest pain, and hemoptysis [3].

The acute surgical abdomen is a condition characterized by the sudden onset of intense, localized, or diffuse abdominal pain; the persistence of the symptoms ranges from hours to days, and it is one of the most frequent reasons for outpatient presentations in the emergency departments worldwide [4]. Due to its varying clinical presentation, diagnosis poses a real challenge for emergency physicians around the world [5]. The vast array of underlying causes makes it a time-sensitive condition that must be rapidly diagnosed and treated to avoid severe complications, such as sepsis, necrosis, gangrene, and fistula, leading to death. According to literature data, over 6% of acute surgical abdomens are misdiagnosed [4]. Some of the most prevalent pathologies which can be misdiagnosed as an acute surgical abdomen are acute inferior wall myocardial infarction, with a prevalence of 40% and Diabetic Ketoacidosis, with a prevalence of 65% among type I diabetic patients, followed by Sickle Cell Crysis, Pneumonia, Reno-ureteral cholic, Herpes Zoster infection, Irritable Bowel Syndrome, Pelvic Inflammatory Disease, and many more [6]. Previous research has shown that accurate pre- and perioperative diagnosis can dramatically reduce management errors, with lower rates of unnecessary surgical interventions and perioperative complications. Also, the extensive and confirmatory pre-operative diagnosis of acute abdomen ensures optimal decision-making for surgical treatment, hence reducing the incidence of negative laparotomies [7].

The literature suggests that severe COVID-19 increases the risk of acute abdominal pathologies, such as pancreatitis, appendicitis, and cholitis, likely due to inflammation. However, the underlying mechanisms remain unclear [8,9].

The aim of the current study is to provide a better understanding and overview of the involvement of SARS-CoV-2 infection in the pathophysiological mechanism of the acute surgical abdomen.

We performed an extensive literature search using the PubMed, Google Scholar, Web of Science, and EBSCO databases, using the keywords “acute surgical abdomen”, “COVID-19”, “inflammation”, “cytokine storm”, and “thrombosis”.

## 2. Coronavirus Disease 19

Coronavirus disease 2019 is a viral infection with a high level of contagiousness and various transmission pathways. Produced by SARS-CoV-2, it is estimated to have produced between 6 and 18 million deaths worldwide and has had devastating effects around the globe, hence the need for the declaration of a global pandemic by the WHO in early 2020 [10,11].

The COVID-19 pandemic has caused an unparalleled worldwide crisis, forcing many countries to impose curbs on population travel to limit the spread of SARS-CoV-2 and protect health services from becoming overwhelmed [12].

Significant disparities across nations and between genders were observed in both the number of cases and deaths, where data on gender and age categories were available. In almost all countries, the mortality rate for men was significantly higher in all age groups, particularly among younger people. However, a simple analysis of 20 European nations, as well as the United States and Canada, clearly suggests that the death rates of COVID-19, regardless of the gender, appear to be primarily determined by the proportion of patients diagnosed with SARS-CoV 2 who are over the age of 70–75 [13].

Studies have also shown that patients with underlying chronic diseases, such as cardiovascular pathology, diabetes, cancer (in particular pulmonary), chronic obstructive pulmonary disease, and arterial hypertension, have a more elevated risk for developing severe forms of the disease, hence the occurrence of serious complications [14].

Respiratory droplets and aerosols are the main transmission port of the infection among humans. Once within the body, the virus connects to host receptors before entering host cells via endocytosis [15]. The spike protein (S) is a crucial determinant of cell tropism and thus interspecies transmission of SARS-CoV-2, binding the virus to a cellular receptor and then catalyzing virus entrance via membrane fusion [16]. The primary host receptor for the penetration into cells is Angiotensin Converting Enzyme 2 (ACE-2), which is extensively expressed in adult nasal epithelial cells. The virus replicates and propagates locally, infecting ciliated cells in the conducting airways, followed by virus migration from the nasal epithelium to the upper respiratory tract [17]. The virus invades type 2 alveolar epithelial cells by the aid of the host receptor ACE-2, where it begins to replicate to yield additional viral nucleocapsids. Pneumocytes infected with the virus produce many cytokines and inflammatory markers, including interleukins 1, 6, 8, 120, and 12, as well as Tumor Necrosis Factor-α (TNF-α), Interferon-λ (IFN-λ), and Interferon-β (IFN-β), Chemokine interferon-γ inducible protein 10 (CXCL-10), Monocyte chemoattractant protein-1 (MCP-1), and Macrophage inflammatory protein-1 alpha (MIP-1α). Chemotaxis draws neutrophils, CD4 helper T cells, and CD8 cytotoxic T cells into the lung tissue, causing further inflammation and extensive lung injury. Through apoptosis, the host cell releases viral particles that infect the nearby type 2 alveolar epithelial cells. The persistent damage is caused by the secluded inflammatory cells and continuous viral replication. This results in the destruction of both type 1 and type 2 pneumocytes, the consequence being extensive alveolar damage. Ultimately, this leads to acute respiratory distress syndrome [17].

While most patients with mild to moderate disease first presented fever, cough, headache, muscular soreness, anosmia, and dyspnea are among the symptoms of COVID-19, some patients demonstrated unusual symptoms, such as diarrhea and vomiting [15]. Severe acute respiratory infection symptoms appeared early in the course of this pneumonia, with some patients rapidly developing acute respiratory distress syndrome (ARDS), abrupt respiratory failure, often leading to death, despite proper treatment [18].

The ACE2 is extensively disseminated in several organs and tissues, which correlates with the vast array of clinical systemic symptoms and multiorgan dysfunction seen with COVID-19, such as respiratory, renal, hepatic, gastrointestinal, and cardiovascular [19].

Another serious consequence of COVID-19 infection is represented by the initiation of a hypercoagulable state. Elevated levels of fibrinogen, von Willebrand factor (VWF), and the fibrin degradation product D-dimer in the blood indicate the presence of a COVID-19-specific coagulopathy. Patients exhibit minor or no changes in prothrombin time, activated partial thromboplastin time, antithrombin levels, activated protein C levels, and platelet count. These characteristics are more consistent with a state of hypercoagulability combined with a severe inflammatory condition [20].

Coronavirus-induced coagulopathy occurs through a variety of pathophysiological mechanisms triggered by the “cytokine storm,” including systemic inflammation caused by the activation of Toll-like receptor, endothelial dysfunction due to an increase in intracellular Von Willebrand factor and the initiation of the intrinsic coagulation cascade, via tissue factor release promoted by a procoagulant state [21]. These mechanisms appear to respect Virchow’s triad of extensive endothelial cell damage, blood flow dynamics alteration, and abnormal platelet activation [18].

The immune system’s role is to detect harmful agents in the body and initiate an inflammatory response aimed at suppressing them. This process also aids in damage repair before returning to a physiological state. Cytokines play a crucial role in this coordination, acting as a communication system among immune cells. A sophisticated network of regulatory mechanisms ensures a balance between pro-inflammatory and anti-inflammatory cytokines, keeping the immune response appropriate to the level of threat and allowing it to subside once the threat is abolished. If any of these mechanisms fail, it can result in excessive immune activation and an overproduction of cytokines, leading to a systemic inflammatory response that can have detrimental effects on the entire organism, also known as a cytokine storm [22].

Increased levels of cytokines lead to endothelial dysfunction, vascular injury, and metabolic imbalances, resulting in damage to various organ systems [23]. TNF-α, interleukin 1, 2, 6, and 10, IFN-γ-inducible protein 10 (IP-10), MCP-1, and Granulocyte Macrophage-Colony Stimulating Factor (GM-CSF) are among the most prevalent proinflammatory cytokines identified as being increased in a COVID-19-induced cytokine storm [24]. A retrospective, multicentric study has identified elevated ferritin and interleukin 6 levels, which may be a predictor of mortality among COVID-19-infected patients [25].

The syndrome can present itself with flu-like symptoms, which can evolve to disseminated intravascular coagulation (DIC), hypoxemia, shock, and hemostasis imbalances, culminating in death [26].

## 3. Acute Surgical Abdomen

The acute surgical abdomen is a life-threatening condition that requires immediate care and treatment, usually caused by inflammation, infection, ischemia, intrinsic or extrinsic obstruction, and traumatic events [27]. As a general rule, acute surgical abdomen is consistent with sudden severe abdominal pain, with a rapid evolutive pattern, caused by an intraabdominal pathology, requiring immediate surgical intervention [28].

The usual clinical presentation is abdominal pain associated with a various array of symptoms like nausea, vomiting, bloating, fever, constipation, inappetence, diarrhea, abdominal distension, jaundice, or even shock [29,30].

Abdominal pain accounts for from about 5 to 10% of all emergency department (ED) visits worldwide; in the United States, the number of presentations reaching as high as 3 million patients per year [31,32], out of which from 20 to 25% are diagnosed with a serious intra-abdominal condition requiring hospitalization and immediate surgical intervention [33]. According to current studies, there is no significant difference in the incidence and prevalence of abdominal pain and the occurrence of acute surgical abdomen related to gender [34].

Diagnostics are, nevertheless, a challenge for any physician due to the vast symptoms at presentation, many of which are nonspecific, hence the need for a thorough history taking, clinical examination, paraclinical investigations, and extensive differential diagnosis [33,35].

The most common causes of acute abdominal pain are represented by non-specific abdominal pain, reno-ureteral colic, acute appendicitis, biliary disorders, diverticulitis, bowel obstruction, acute pancreatitis, gastritis, gastroenteritis, inflammatory bowel disease, mesenteric infarction, gynecological disorders, and others [34].

Appendicitis is less common in people over 50 years old, while cholecystitis, bowel obstruction, perforated malignancies, strangulated hernias, and acute mesenteric ischemia are more prevalent [36].

In Asia, findings reveal a high number of perforated gastric tumors, predominantly among the elderly. Ileal perforation as a complication of Typhoid fever, tuberculous peritonitis, obstructive biliary and enteric ascariasis, amoebic liver abscess, and other less frequent abdominal parasitosis are prevalent in endemic tropical and subtropical areas, particularly in low- and middle-income countries. Moreover, high-income countries with an older population and a high-fat, low-fiber diet have a greater incidence of complicated diverticulitis and colon-rectal neoplasia; hence, estimations that around 49 million general surgical emergency procedures are conducted each year worldwide [36].

The range of the mortality rates for all adults with an acute abdomen is 2 to 14%, depending mainly on the primary pathology and age, the elderly being more liable [36]. Moreover, underlying diseases, such as cardiovascular conditions, chronic kidney disease, active malignancies, and the presence of Systemic Inflammatory Response Syndrome (SIRS), sepsis, septic shock, and Multi-System Organ Failure (MSOF) at presentation, were positively correlated with increases in mortality rates [37]. The prediction of the overall outcome in patients with acute surgical abdomen requiring emergency surgical intervention may also be suggested by the increased values of the serum lactate, interleukin 6, C-Reactive Protein, and procalcitonin levels at presentation and their peri- and postoperative evolution [38].

Left untreated, acute surgical abdomen can lead to serious complications, such as bleeding, fistulas, peritonitis, necrosis, sepsis, septic shock, exacerbation of preexisting pathologies, and even death [36].

## 4. Pathophysiological Mechanisms of COVID-19-Associated Acute Surgical Abdomen

Beginning with the presumption that the mechanism of action in COVID-19 infections involves the virus’s capacity to attach to angiotensin-converting enzyme 2 (ACE2) receptors, it is hypothesized that the manifestation of multiple organ dysfunction is closely linked to COVID-19, owing to the extensive presence of ACE2 across various organs [39]. The confirmation of this supposition came from gastrointestinal biopsies, which identified the coronavirus RNA in the examined samples. One of the mechanisms is attributed to direct damage induced by the virus and an inflammatory response mediated through immunological pathways, which may lead to malabsorption, malfunction of the intestinal mucosa and disruption of intestinal secretion production, and stimulation of the enteric nervous system [40].

A second incriminated mechanism is represented by the COVID-19-induced coagulopathy, which, unlike other coagulopathies, is characterized by elevated fibrinogen levels and extensive fibrinolysis (elevated D-dimers), but with negligible alterations in platelet count, prothrombin time, and antithrombin levels. The extensive inflammatory response causes endothelial damage, which activates coagulation. This leads to endothelial infiltration by monocellular cells, endothelial inflammation with lymphocytic infiltration, platelet activation, and uncontrolled coagulation [41]. Clot formation is accelerated by the decrease in Plasmin activity and an increase in plasminogen inhibitor activator-1 [42]. As stated by previously published articles, hypoxia, which was present in a vast majority of patients with moderate to severe forms of COVID-19, may enhance the procoagulant state by upregulating the function of Tissue Factor in oxygen-deprived vessels [43].

During the COVID-19 pandemic, it was observed that some patients infected with SARS-CoV-2 only had gastrointestinal symptoms as an initial presentation, without pulmonary involvement, hence the hypothesis that direct injury by the virus may be the main cause [44]. Taking into account the two pathophysiological mechanisms, several articles described cases of acute intraabdominal pathologies related to SARS-CoV-2 2infection.

**Figure 1:**
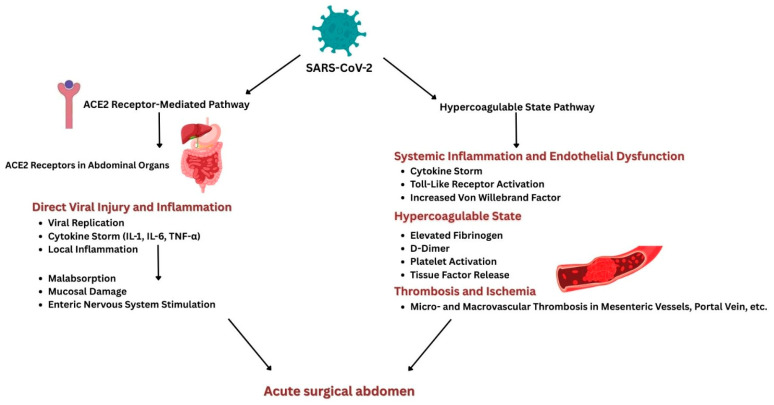
Schematic illustration of pathophysiological pathways connecting COVID-19 and acute surgical abdomen.

The two main pathways by which COVID-19 can lead to an acute surgical abdomen are illustrated in Figure 1.

### 4.1. The ACE2 Receptors Hypothesis

A study published by *Xiao* et al. identified, by immunofluorescent methods, a high expression of ACE2 receptors in the glandular cells of the gastric, duodenal, and rectal epithelium. Moreover, by RNA detection methods, SARS-CoV-2 viral RNA and nucleocapsid proteins were identified in the above-described epithelia [45].

The detection of SARS-CoV-2 in stool was significant, suggesting the virus can multiply and persist in the digestive tract. A meta-analysis conducted by *Cheung* et al. states that the persistent finding of viral RNA in feces indicates that infectious virions are excreted by virus-infected gastrointestinal cells. Nonetheless, the percentage of stool samples positive for viral RNA was around 48%; among these, approximately 70% of samples collected after viral clearance from the respiratory specimens also tested positive for the virus (95% CI, 49.6–85.1) [46]. Several studies have proven that viral infections can lead to acute appendicitis by various mechanisms, such as lymphoid hyperplasia, followed by appendiceal obstruction and ulcerations in the appendix mucosa, followed by a secondary bacterial colonization [47]. According to some studies, cases of acute appendicitis as initial complaints of COVID-19 infection were described in some of the patients as being the only symptoms at presentation [48,49]. Moreover, a case report by *Kono* et al. demonstrated the presence of SARS-CoV-2 RNA in a specimen of phlegmonous appendicitis by RT-PCR techniques, proving an association between the occurrence of acute appendicitis and COVID-19 infection [50]. Furthermore, another study conducted by *Georgakopoulou* et al. sustains the above-mentioned hypothesis by identifying peculiar histological displays, such as sparse microthrombi, fibrinoid necrosis of appendicular vasculature, and lymphocytic infiltrates around the vessels, which are consistent with coronavirus infection [51].

Another study of *Burkett* et al., performed on four patients, identified that the specimens harvested by hemicolectomy from COVID-19-infected patients showed ischemic alterations, acute and chronic inflammation, and fibrinous microthrombosis in small blood vessels in the regions where mucosal ulceration was present. Nonetheless, by means of immunofluorescence and In Situ Hybridization performed on the specimens from the affected bowel areas, the presence of SARS-CoV-2 RNA was established [52]. Cases of acute diffuse colitis, ulcerative colitis, lymphocytic colitis, and other types of Inflammatory Bowel Diseases (IBDs) were described; in all cases, viral RNA was detected by RT-PCR in the stool [53,54,55].

Several patients with COVID-19 infection developed newly diagnosed type I diabetes melitus (DM), hence the hypothesis of the COVID-induced diabetes, which was also confirmed by a metanalysis conducted by *Shrestha* et al., which stated that the prevalence of COVID-induced type I DM is as high as 20%, also implying higher death rates than in patients previously diagnosed with DM [56,57]. This can be attributed to the high extent of ACE2 receptors in the pancreatic tissue, within the pancreatic islets and exocrine glands, which is even higher than in the lung tissue, hence the capacity of SARS-CoV-2 to directly bind to the pancreas, inducing pancreatic damage and subsequent distress [58]. According to *Hadi* et al., pancreatic destruction occurs by direct viral injury of the acinar cells due to inflammation and edema, while another theory is that, by injuring the acinar cells, the leakage of pancreatic enzymes leads to autodigestion of the pancreas [59]. *Hegi* et al. identified that increased cytokine levels, especially those of IL-6, 8, and 10, were present in both severe cases of COVID-19 and acute pancreatitis, explaining the effect of the cytokine storm in the development of acute pancreatitis [60]. A similar theory is sustained by *Wang* et al., who state that the ontogeny of acute pancreatitis may be caused through an exaggerated systemic response to the Acute Respiratory Distress Syndrome (ARDS) or by the cytokine storm induced by the COVID-19 infection, which leads to multisystem organ failure [61]. A literature review conducted by *Jabłońska* et al. revealed a considerably greater incidence of “idiopathic” AP in patients infected with COVID-19 than in non-COVID-19 individuals, hence the conclusion that SARS-CoV-2 may represent a novel infectious element for the development of acute pancreatitis [62].

Nevertheless, hepatic injury was described in several studies, while the primary identified histological abnormalities were the lymphocytic infiltration of the sinusoidal vessels and their dilation, hepatic steatosis, and extensive liver necrosis assigned to direct viral injury, due to the high expression of ACE2 receptors [63]. Also, some studies correlate the presence of liver involvement with the severity of COVID-19 infection [64].

### 4.2. The Hypercoagulable State Hypothesis

As stated by *Avila* et al., previous research has explored the involvement of inflammation in causing a hypercoagulable status, probably due to stimulation of endothelial cells, platelets, and leukocytes, inducing tissue factor (TF), which, by binding to clotting factor VIIa, initiates the coagulation process [65]. A study conducted by *Lippi* et al. determined that the hypercoagulable state in COVID-19 is dominated by the elements of Disseminated Intravascular Coagulation (DIC), such as elevated D-Dimer values, prolonged prothrombin time, and hyperfibrinogenemia [66].

A potential mechanism proposed by *Abou-Ismail* et al. states that the production of proinflammatory cytokines may enhance epithelial cells and monocyte and macrophage activity, which, superimposed with the activation of endothelial cells by direct infection through the ACE2 receptors, leads to the formation of the fibrin clot by thrombin overproduction. Moreover, this mechanism is enhanced by the overproduction of Tissue Factor (TF), activation of thrombocytes, and increased production of Von Willebrand Factor and coagulation factor VIII.

Further on, thrombin sustains inflammation by triggering platelet activation, which further leads to the production of Neutrophil Extracellular Traps [67].

Considering these mechanisms, several case reports have described cases of ischemia and thrombosis in different intraabdominal organs and vessels, both microvascular and large vessels, as a consequence of concomitant or recent COVID-19 infection.

Several cases of abdominal thromboembolic complications of COVID-19 infection have been reported, as shown in Table 1. All patients included in these studies had a positive RT-PCR test for COVID-19 previously (maximum 14 days prior) or concomitant with the current presentation.

## 5. Discussions and Limitations

While existing literature has explored the individual aspects of COVID-19 and acute surgical abdomen, this review distinguishes itself by providing a comprehensive and integrated analysis of the pathophysiological mechanisms linking the two. Unlike previous studies that primarily focus on specific complications or individual case reports, our work synthesizes the current understanding of how COVID-19-induced inflammation, hypercoagulability, and ACE2 receptor involvement contribute to the development of various acute surgical abdominal pathologies. Furthermore, this review offers a consolidated overview of the thromboembolic complications associated with COVID-19 in the context of acute surgical abdomen, providing a valuable resource for clinicians and researchers seeking a holistic understanding of this complex interplay. The present work must also be viewed in light of some limitations. The investigation was limited to literature published in English, which could result in bias by excluding relevant studies published in other languages. The selection of studies for inclusion in the present review involved a degree of subjective judgment based on the inclusion and exclusion criteria. Furthermore, the included studies may have varied significantly in terms of study design, patient populations, interventions, and outcome measures. This heterogeneity could limit the ability to draw firm conclusions or make strong recommendations.

## 6. Conclusions

Infection with the novel coronavirus may have a major role in the pathophysiological mechanism of the acute surgical abdomen, mainly through the two described mechanisms involving the ACE2 inhibitors widely spread into the gastrointestinal tract and the thrombogenic effects leading to vessel occlusion. Thus, further studies are needed to elucidate the complex pathophysiological mechanism leading to acute abdominal pathology and whether the abdominal complications are directly related to COVID-19 infection.

## Figures and Tables

**Table 1:** Case reports of thromboembolic complications of COVID-19 infection.

Author	Country	Age	Sex	Initial Symptoms	Thrombotic Complication	Intraoperatory Findings
*Borazjani, R* et al. *[68]*	Iran	23 years	M	Fever, hemoptysis, diarrhea, nausea, vomiting	Acute portal vein thrombosis	N.A
*Cheung S* et al. *[69]*	USA	55 years	M	Abdominal pain, nausea, diarrhea	Superior mesenteric artery occlusion	Ischemic small bowel
*Posada-Arango AM* et al. *[70]*	Peru	62 years	M	Colicative abdominal pain, emesis	Superior mesenteric artery thrombosis	Necrosis and segmental ischemia throughout the jejunum and ileum
		22 years	F	Mezogastric abdominal pain	Acute/subacute thrombosis of mesenteric vein, splenic infarction	N.A
		65 years	F	Upper abdominal pain	Left jejunal artery thrombosis	Intraperitoneal collection, segmental ischemia of jejunum
*Badrawi N* et al. *[71]*	UAE	41 years	F	Colicative right lower abdominal pain, vomiting, dysuria, vaginal discharge, weight loss	Right ovarian vein thrombosis	N.A
*Baeza C* et al. *[72]*	Spain	63 years	F	Pain in the lower limbs	Thrombosis of the infrarenal aorta with extension through both iliac axes	Thrombosis of the infrarenal aorta with extension through both iliac axes
		69 years	M	Severe pain in the lower left limb, impaired mobility and lack of sensitivity	Occlusion of the distal abdominal aorta and the origin of both common iliacs	Occlusion of the distal abdominal aorta and the origin of both common iliacs
		85 years	F	Gluteal pain radiating to both lower limbs and numbness	Occlusion in the aortic bifurcation and both common iliac arteries	Occlusion in the aortic bifurcation and both common iliac arteries
*Segovia FD* et al. *[73]*	USA	64 years	F	Abdominal pain, distension, constipation	Superior mesenteric artery occlusion	Ischemia in the vascularization area of the superior mesenteric artery and significant colon ischemia
*Hanif M* et al. *[74]*	Pakistan	20 years	F	Abdominal pain and abdominal distension	Superior mesenteric artery occlusion	Gangrene of the whole of the small gut, except proximal 3 feet from the duodenojejunal junction
*Morioka, H* et al. *[75]*	Japan	64 years	M	Abdominal pain	Thrombosis in the aorta, perforative peritonitis due to ileal ischemic necrosis	perforation due to necrosis at multiple sites in the ileum
*de Barry O* et al. *[76]*	France	79 years	F	Fever, epigastric pain, diarrhea	Right-portal vein thrombosis, thrombosis of the distal part of the upper mesenteric vein extended to the spleno-mesaraic trunk, thrombosis of the upper mesenteric artery and jejunal artery, bowel ischemia of the caecum and small intestine	Necrotic ileum and right colon
*Levolger S* et al. *[77]*	Netherlands	58 years	M	Dispneea, abdominal pain, abdominal distension	Superior mesenteric artery thrombosis, renal and splenic infarction	Small bowel ischemia
*Sinz S* et al. *[78]*	Switzerland	38 years	M	Severe abdominal pain in the epigastrium accompanied by nausea and diarrhea, fever	Extensive portal vein thrombosis, mesenteric venous stasis	N.A
*Rodriguez-Nakamura RM* et al. *[79]*	Mexico	45 years	M	Severe mesogastric pain, fever, nausea, diaphoresis	Mesenteric ischemia	Hemoperitoneum, hypoxic-ischemic changes in the distal ileum and the cecum with necrotic bowel loops
		42 years	F	Abdominal pain and distension, rectorrhagia	Thrombosis of the portal and mesenteric veins	Fecal peritonitis, necrosis of the epiploon, and a jejunal perforation
*Alemán W* et al. *[80]*	Ecuador	44 years	M	Severe abdominopelvic pain, weight loss	Thrombosis of the superior mesenteric, splenic, and portal vein.	N.A
*Philipponnet C* et al. *[81]*	France	52 years	M	Abdominal pain, asthenia	Thrombosis of the left renal artery with extensive ischaemical lesions of the renal parenchyma	N.A
*Qasim Agha O* et al. *[82]*	USA	60 years	M	Moderate, dull, and left-sided abdominal pain	Acute splenic artery thrombosis and infarction of more 50% of the splenic volume	N.A
*Carmo Filho A *[83]**	Brazil	33 years	M	Severe low back pain radiating to the hypogastric region	Inferior mesenteric vein thrombosis	N.A
*Sarkardeh M* et al. *[84]*	Iran	58 years	M	Generalized abdominal pain	Superior mesenteric artery occlusion	Ileal ischemia
		51 years	M	Abdominal pain, fever	Superior mesenteric artery occlusion	Pneumoperitoneum, necrosis and perforation in three areas of the ileum
		60 years	M	Abdominal pain, no intestinal movements (gas or stool)	Superior mesenteric and colic artery occlusion	Pneumoperitoneum, necrosis of the ileum and whole colon
		88 years	M	Abdominal pain, bloating, melena, absent intestinal movements (gas or stool)	Superior mesenteric artery occlusion	Ileal ischemia
*Hashim Z* et al. *[85]*	India	49 years	M	Left upper quadrant abdominal pain	Aortic thrombus and splenic infarct	N.A
		30 years	M	Abdominal pain	Jejunal artery occlusion	Ischemic jejunal perforation with peritonitis
*Voci D* et al. *[86]*	Switzerland	57 years	M	Lower-back and abdominal pain, nausea	Proximal splenic artery stenosis, splenic infarctions, bilateral kidney infarction, dissection of the mesenteric artery	N.A
*Veyseh M* et al. *[87]*	USA	52 years	F	Sharp right upper quadrant abdominal	Ovarian vein thrombosis, which extended partially to the renal vein	N.A
*Antunes de Brito CA* et al. *[88]*	Brazil	45 years	F	Acute abdominal pain	Partial thrombosis in the common hepatic artery	N.A
*Del Hoyo J* et al. *[89]*	Spain	61 years	F	Severe acute abdominal pain and vomiting	Right hepatic vein thrombosis and a complete thrombosis of the splenoportal axis	N.A
*Besutti G* et al. *[90]*	Italy	54 years	M	Sharp right flank and lumbar pain, fever, and dysuria	Large right kidney arterial infarction	N.A
		53 years	M	Severe left flank pain	Large infarcted areas involving the spleen and the left kidney	N.A
		72 years	M	Severe abdominal pain	Thrombosis of superior mesenteric and thoracic aorta	Small bowel ischemia associated with massive splenic infarction
*Azouz E* et al. *[91]*	France	56 years	M	Abdominal pain and vomiting	floating thrombus of the aortic arch and occlusion of the superior mesenteric artery	Ischemic small bowel

M—Male, F—Female, N.A—not applicable, USA—United States of America, UAE—United Arab Emirates.
